# A constrained multinomial Probit route choice model in the metro network: Formulation, estimation and application

**DOI:** 10.1371/journal.pone.0178789

**Published:** 2017-06-07

**Authors:** Yongsheng Zhang, Enjian Yao, Heng Wei, Kangning Zheng

**Affiliations:** 1 School of Traffic and Transportation, Beijing Jiaotong University, Beijing, China; 2 MOE Key Laboratory for Urban Transportation Complex Systems Theory and Technology, Beijing Jiaotong University, Beijing, China; 3 Advanced Research in Transportation Engineering and Systems (ART-EngineS) Laboratory, College of Engineering and Applied Science, The University of Cincinnati, Cincinnati, United States of America; 4 School of Traffic and Transportation, Beijing Jiaotong University, Beijing, China; Beihang University, CHINA

## Abstract

Considering that metro network expansion brings us with more alternative routes, it is attractive to integrate the impacts of routes set and the interdependency among alternative routes on route choice probability into route choice modeling. Therefore, the formulation, estimation and application of a constrained multinomial probit (CMNP) route choice model in the metro network are carried out in this paper. The utility function is formulated as three components: the compensatory component is a function of influencing factors; the non-compensatory component measures the impacts of routes set on utility; following a multivariate normal distribution, the covariance of error component is structured into three parts, representing the correlation among routes, the transfer variance of route, and the unobserved variance respectively. Considering multidimensional integrals of the multivariate normal probability density function, the CMNP model is rewritten as Hierarchical Bayes formula and M-H sampling algorithm based Monte Carlo Markov Chain approach is constructed to estimate all parameters. Based on Guangzhou Metro data, reliable estimation results are gained. Furthermore, the proposed CMNP model also shows a good forecasting performance for the route choice probabilities calculation and a good application performance for transfer flow volume prediction.

## Introduction

With new lines put into operation almost every year, the large scaled metro system has formed in some major cities in China, such as Beijing, Shanghai, Guangzhou and Shenzhen. Taking Guangzhou Metro for instance, up to the year of 2014, it is the sixth busiest metro system in the world and the third largest metro network in China with 9 lines, 164 stations including 21 transfer stations, and 260.5 km of tracks. In a large scaled metro network, the large number of transfer stations which brings plenty of routes for some origin-destination (OD) pairs increases the complexity of route choice modeling. Usually, according to a specific scheme, an individual chooses the best route among many alternative routes with comprehensive consideration of multiple factors, including the variables denoting the level of service of metro system, such as in-vehicle travel time, number of transfers, transfer time, congestion level, etc. and the variables describing the influence of topological structure of the metro network and route direction on passengers’ route choice preferences, such as angular cost [1–2].

**Fig 1:**
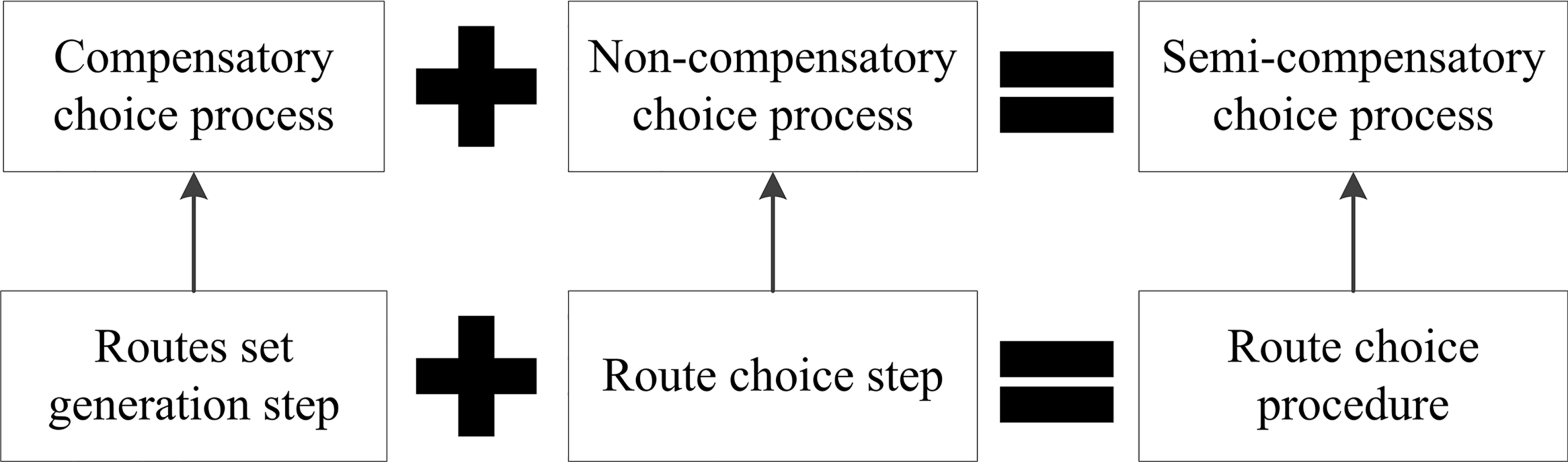
The relationships of different choice processes. In the figure, ‘A+B = C’ means C is the combination of A and B. The arrow displays the one-to-one match.

The complex nature of route choice process responses to a large scaled metro system has brought challenges in establishing route choice model to reveal realistic behavioral decisions in the actual route choice process. Traditionally, with respect to route choice in a large scaled metro network, a route is chosen from a route set which is derived from attributes’ limitations, such as travel time and number of transfers. For example, for an OD pair, if the shortest travel time of one route is 30min, it is a common sense that passengers will not consider the route with more than 60min travel time. In this case, the 60min travel time is the limitation and the routes with less than 60min travel time constitutes the routes set. However, route choice and routes set steps are usually carried out separately and independently in the metro system which leads to losing the consistence between the two steps. For the two steps, the route choice step is a compensatory choice process which focuses on calculating the trade-offs among multiple influencing factors and the routes set generation step is a non-compensatory process which pays attention to the cut-offs associated with the attributes’ limitations. The non-compensatory behavior has been proved in the choice process [3–5]. And semi-compensatory route choice modelling which combines the routes set generation and route choice steps has attracted much more attention. The relationships between semi-compensatory, compensatory and non-compensatory choice processes are shown in Fig 1.

Meanwhile, route over-lapping problem in the large scaled metro network has already been figured out by Yai et al. [6]. Especially for some OD pairs with long direct distance, the fact that some alternative routes will share some links brings the correlation among the routes. Although most Logit-based models were satisfactory in representing route choice behavior associated with route over-lapping problem, they were still the approximate responses to the real behavior. In order to exactly express the interdependency among alternatives, Probit model [7] is more suitable though its estimation is a little harder than Logit models. Faced with elaborative operational requirements and services, the operational department is looking forward to a more advanced route choice model to reveal passengers’ actual route choice behaviors so as to support personalized travel service and travel demand prediction.

Therefore, it is necessary to establish a semi-compensatory Probit route choice model and design an easier estimation approach for practice. In this paper, a constrained multinomial Probit route choice model is proposed to reveal the realistic route choice process along with the estimation approach, focusing on analyzing the semi-compensatory choice behavior and representing the interdependency among alternative routes.

## Literature review

Route choice model based on random utility maximization (RUM) theory [8] mainly consists of two types, including Logit and Probit models. Among various models, Multinomial Logit (MNL) model [9] is the most widely used due to its easy estimation and application. Ramming, Raveau et al., Zhang et al. and Liu et al. successfully analyzed route choice behavior with the consideration of level of service, social demographics, travel purpose and route direction based on MNL model [1, 2, 10–11]. But the assumption that the error component follows an identical and independent (IID) Gumbel distribution induces many weaknesses. In order to alleviate one of the weakness, known as route over-lapping problem which is caused by the interdependency among routes, many extended Logit models are developed, such as C-Logit [12], Path Sized Logit (PSL) [13–14], Paired Combinatorial Logit (PCL) [15–16], Cross Nested Logit (CNL) [17], Generalized Nested Logit (GNL) [18], Mixed Logit [19], etc. For the application in the metro network, Raveau et al. applied successfully C-Logit model to analyze passengers’ route choice preferences [20]. However, Logit models cannot avoid IID distribution assumption to formulate probability equation with closed form, weakening the interdependency among alternative routes, while Probit model [7] can reflect deeply the interdependency by covariance, closer to passenger’s actual route choice behavior. Yai et al. proposed a Probit model with structured variance to analyze route choice behavior in the railway network, saving the computational time to an extent [6].

Those models mainly focus on the process that an individual chooses the best route from a given routes set. The consistence between the routes set generation and route choice processes is usually neglected for metro passengers. Considering the interplay between the two sub-processes, Zhang et al. [21] successfully introduced constrained multinomial logit (CMNL) model [22–23] into route choice modeling in the metro network to analyze passengers’ semi-compensatory choice behavior. Semi-compensatory models combining compensatory and non-compensatory behaviors have been paid more and more attention [24–26]. As one of two major approaches, the two-stage approach is widely used, consisting of two stages: generating all possible consideration routes sets and then choosing routes from the generated routes sets [27]. The consideration routes set is a subset of master routes set which is limited by some specific attributes. The two-stage approach is attractive that different models are allowed to explain each stage and many successful applications have already been found in the literature [28–30]. However, it leads to computational complex because too many consideration routes sets need to be constructed from master routes set [31]. And it also would have no sufficient robustness of choice prediction at the level of individual sets [32]. In order to avoid such a complex combinatorial number of choice sets, a kink called non-compensatory component is added to utility function, known as the second semi-compensatory choice modeling approach [33–34]. The non-compensatory component won’t affect the utility when the attribute value lies in the domain, while it will negatively and significantly affect the utility when the attribute value exceeds the threshold. The non-compensatory component simplifies the structure of semi-compensatory choice model and saves the computational time by avoiding huge number of consideration choice sets compared with the two-stage approach. However, in those researches, kinks in the utility function make it non-differentiable at the cut-off, which is difficult to be applied in equilibrium and optimization processes.

To solve this problem, a constrained multinomial logit (CMNL) route choice model [21] is developed where the non-compensatory component is a continuous function, making utility function differentiable at the cutoff. However, its error component still follows (IID) Gumbel distribution and the route over-lapping problem in the route choice context still needs to be solved. Moreover, compared with the Gumbel distribution, the normal distribution is more approximate to the actual distribution of error component. To address the aforementioned problems, a study for developing a constrained multinomial probit (CMNP) route choice model for metro passengers is proposed in the paper. In this model, the error component follows the normal distribution instead of the IID Gumbel distribution to avoid the weaknesses. The correlations among alternative routes are measured by the covariance matrix.

The following sections of this paper are organized as follows: Section 3 introduces the CMNP route choice modeling methodology; Section 4 is about the estimation approach which is carried out based on MCMC method after transforming the CMNP model into Bayesian formulation; in Section 5, the CMNP model is estimated by the proposed estimation approach based on surveyed RP data in Guangzhou Metro and applied in forecasting the transfer passengers volumes; Section 6 is the conclusions.

## Modeling methodology

Based on random utility theory, the utility function with constrained characteristic attributes mainly consists of three parts: compensatory, non-compensatory and error components.

$$U_{n,k}^{rs}=V_{n,k}^{rs}+C_{n,k}^{rs}+\varepsilon_{n,k}^{rs}$$ (1)

where with respect to route *k* for OD pair *rs*, $U_{n,k}^{rs}$ is the generalized utility perceived by passenger *n*; $V_{n,k}^{rs}$ is the compensatory component; $C_{n,k}^{rs}$ is non-compensatory component; $\varepsilon_{n,k}^{rs}$ is the random error component.

The compensatory component $V_{n,k}^{rs}$ is a trade-off function of characteristic attributes, including level of service variables, network topology, etc. This function represents the compensatory trade-offs among attributes. For simplicity, the compensatory component is defined as a linear function of attributes as shown below.

$$V_{n,k}^{rs}=\sum_{h=1}^{H}\theta_{n,h}X_{k,h}$$ (2)

where *H* is the number of characteristic attributes; *X*_*k*,*h*_ denotes the attributes; *θ*_*n*,*h*_ is the corresponding parameters needed to be estimated.

The non-compensatory component $C_{n,k}^{rs}$ is a cut-off function which should satisfy below conditions:

1. It should be a continuous function which guarantees the application in traffic equilibrium and optimization process.
2. If a constrained attribute value of one route exceeds threshold, non-compensatory component will let the route utility tend to be negative infinity. Otherwise, non-compensatory component tends to be zero.

Therefore, the non-compensatory component $C_{n,k}^{rs}$ is formulated in this paper as follows:

$$C_{n,k}^{rs}=\text{ln}\phi_{n,k}^{rs}$$ (3)

where $\phi_{n,k}^{rs}$ is a continuous function limited in (0, 1). The part $\phi_{n,k}^{rs}$ can be formulated as a probability function which represents the probability that route *k* is considered after the comparison between the constrained attributes of route *k* and the corresponding thresholds. Usually, more than one attribute is the constraint, according to conjunctive screen rule [35], $\phi_{n,k}^{rs}$ can be defined as follows:

$$\phi_{n,k}^{rs}=\prod_{i=1}^{I_{n}}\varphi_{n,k}^{rs}(X_{k,i}^{rs})$$ (4)

where for OD pair *r-s*, $X_{k,i}^{rs}$ is the constrained attribute *i* of route *k*; *I*_*n*_ is the number of attributes constrained by individual *n*; the function $\varphi_{n,k}^{rs}(X_{k,i}^{rs})$ measures the considered probability influenced only by characteristic attribute $X_{k,i}^{rs}$. To specify this function, we can assume a scenario that a constrained attribute $X_{k,i}^{rs}$ with perceived error *Ψ*_1_ has an upper bound $b_{n,i}^{rs}$ with perceived error *Ψ*_2_ (if it is a lower bound, the sign will reverse), then the function $\varphi_{n,k}^{rs}(X_{k,i}^{rs})$ can be calculated by

$$\begin{array}{ll} \varphi_{n,k}^{rs}(X_{k,i}^{rs}) & =P(X_{k,i}^{rs}+\psi_{1}\leq b_{n,i}^{rs}+\psi_{2})\\ & =P(\psi_{1}-\psi_{2}\leq b_{n,i}^{rs}-X_{k,i}^{rs}) \end{array}$$ (5)

where for OD pair *r-s*, $b_{n,i}^{rs}$ is the threshold of attribute *i* constrained for individual *n*.

**Fig 2:**
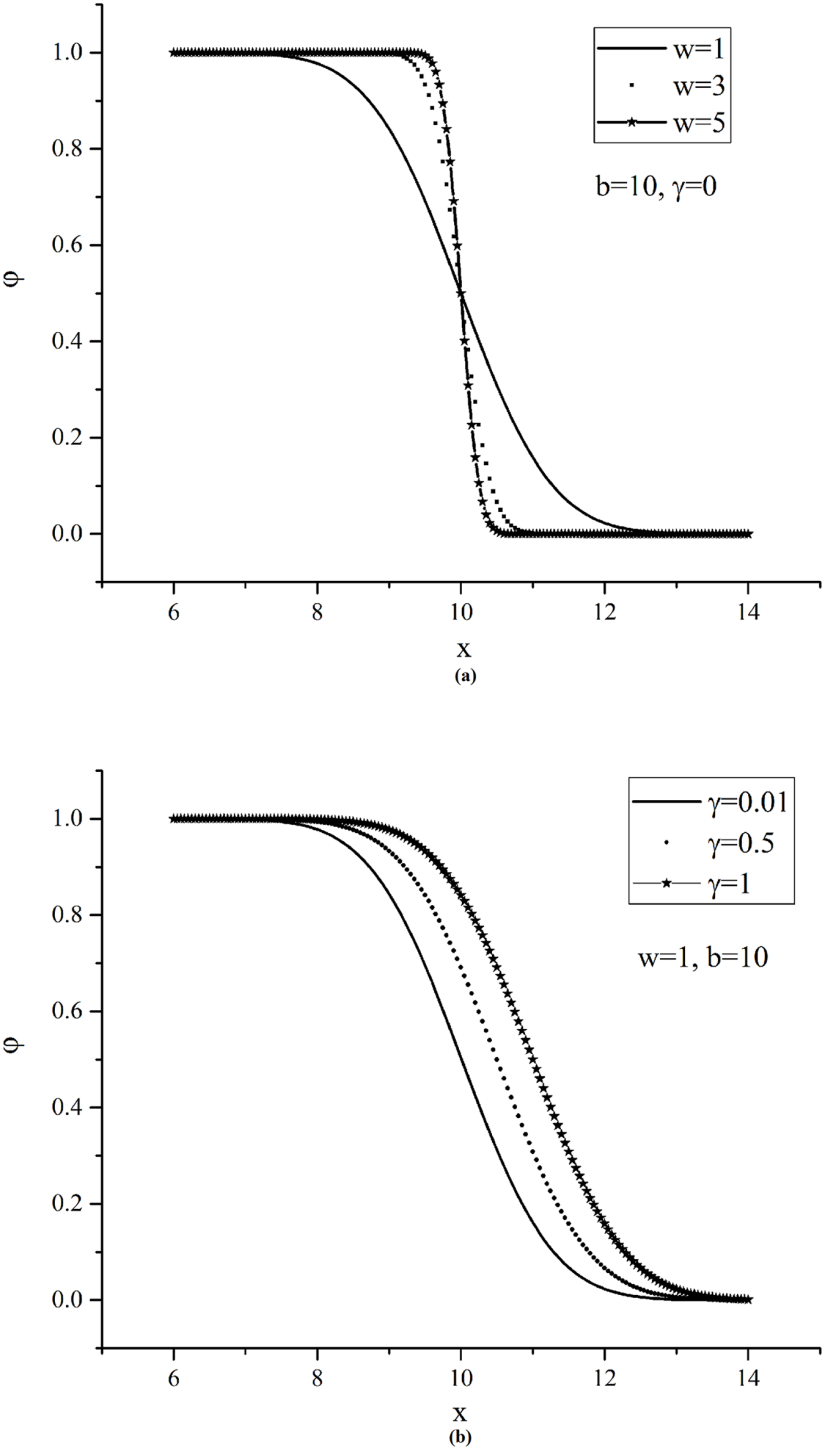
The impacts of scale and location parameters. The impacts of scale and location parameters on considered probability function are displayed separately by changing the values.

In this paper, we assume that the perception errors of the constrained attribute and threshold both follow the normal distributions and are independent from each other. Further, the errors *Ψ*_1_ and *Ψ*_2_ respectively follow normal distributions $\psi_{1}\sim N(0,\sigma_{1}^{2})$ and $\psi_{2}\sim N(\gamma_{i},\sigma_{2}^{2})$, where $\sigma_{1}^{2}$ and $\sigma_{2}^{2}$ are the variances, and *γ*_*i*_ is the mean denoting the location parameter. According to the property of normal distribution, *Ψ*_1_-*Ψ*_2_ still follows normal distribution, that is *Ψ*_1_-*Ψ*_2_ ~*N*(−*γ*_*i*_, σ^2^), where $\sigma^{2}=\sigma_{1}^{2}\text{+}\sigma_{2}^{2}$. And the function $\varphi_{n,k}^{rs}(X_{k,i}^{rs})$ is equal to

$$\begin{array}{ll} \varphi_{n,k}^{rs}(X_{k,i}^{rs}) & =\Phi [\frac{b_{n,i}^{rs}-X_{k,i}^{rs}+\gamma_{i}}{\sigma }]\\ & =\Phi [\omega_{i}\cdot (b_{n,i}^{rs}-X_{k,i}^{rs}+\gamma_{i})] \end{array}$$ (6)

where *ω*_*i*_ is the scale parameter related to the variance (*ω*_*i*_>0, if $b_{n,i}^{rs}$ is the upper bound of $X_{k,i}^{rs}$; else, *ω*_*i*_<0) which affects the changing speed of the probability from 0 to 1; Φ(·) is the cumulative probability function of standard normal distribution. On account of the location parameter *γ*_*i*_, even if the constrained characteristic attribute value is equal to the threshold, the considered probability may not be 0.5, depending on individual preference. The impacts of these parameters (e.g. the scale parameter *ω*_*i*_ and location parameter *γ*_*i*_) on the function are shown in Fig 2.

Usually, in the metro system, the spatiotemporal constraints, referring to travel time and number of transfers, are taken as the constrained characteristic attributes. The fact that the passengers generally prefer to the route with smaller values of the two attributes leads to the result that both of the two constraints only have the upper bounds. In the route choice context for metro passengers, the thresholds to a specific attribute vary with OD pairs. The deterministic parts of the thresholds of constrained travel time and number of transfers are shown below respectively.

$$b_{n,t}^{rs}=\alpha_{n}\cdot \text{ln}(T_{\text{min}}^{rs}\text{+1})+T_{\text{min}}^{rs}$$ (7)

$$b_{n,\tau }^{rs}=\beta_{n}\text{+}M_{\text{min}}^{rs}$$ (8)

where $b_{n,t}^{rs}$ is the bound of travel time (including in-vehicle time and transfer time) of individual *n* for OD pair *rs*, h; $b_{n,\tau }^{rs}$ is the bound of number of transfers; $T_{min}^{rs}$ is the shortest travel time for OD pair *rs*, h; $M_{min}^{rs}$ is the minimum number of transfers; *α*_*n*_ and *β*_*n*_ are the bound parameters needed to be calibrated.

Assuming that error component $\varepsilon_{n,k}^{rs}$ follows the multivariate normal distribution, that is $\varepsilon_{n,k}^{rs}\sim MVN(0,\Sigma_{n}^{rs})$, where $\Sigma_{n}^{rs}$ is the covariance matrix associated with the correlation among alternative routes, together with the constraints on route availability in the utility function, it is called CMNP (constrained multinomial probit) model. With respect to the route choice scenario, the route over-lapping problem in the railway network has been identified by Yai et al. [6] which is similar to metro network. This paper rewrites the covariance matrix into three parts, where the first part depends on the correlation among routes, the second one denotes the transfer variance of the route, and the last one denotes the unobserved variance. The latter two parts distribute independently by route.

$$\Sigma_{n}^{rs}=\sigma_{L}^{2}\left(\begin{array}{cccc} \delta_{11}^{rs} & \delta_{12}^{rs} & \cdots & \delta_{1m}^{rs}\\ \delta_{21}^{rs} & \delta_{2\text{2}}^{rs} & \cdots & \delta_{2m}^{rs}\\ \cdots & \cdots & \cdots & \cdots \\ \delta_{m1}^{rs} & \delta_{m2}^{rs} & \cdots & \delta_{mm}^{rs} \end{array}\right)+\sigma_{N}^{2}\left(\begin{array}{cccc} X_{12}^{rs} & 0 & \cdots & 0\\ 0 & X_{22}^{rs} & \cdots & 0\\ \cdots & \cdots & \cdots & \cdots \\ 0 & 0 & \cdots & X_{m2}^{rs} \end{array}\right)+\sigma_{0}^{2}I$$ (9)

$$\delta_{kj}^{rs}=\sum_{i\in \Gamma_{k}}\kappa_{ikj}l_{i}$$ (10)

where *m* is the number of alternatives in the routes set; $\sigma_{L}^{2}$ is the unit variance which is independent from each other; $\sigma_{N}^{2}$ is the variance of transfer 1time; $\sigma_{\text{0}}^{\text{2}}$ is constant and identical to all routes; $\delta_{kj}^{rs}$ is the over-lapping length between route *k* and *j* for OD pair *rs*; $X_{m2}^{rs}$ is the number of transfers of route *m*; *I* is the identity matrix; *l*_*i*_ is the length of link *i*; Γ_*k*_ is the links set of route *k*; if link *i* is shared by route *k* and *j*, *k*_*ikj*_ = 1, otherwise, *k*_*ikj*_ = 0. There are only three parameters in this covariance matrix, but we just need to estimate the ratio *λ*_1_ of $\sigma_{L}^{2}$ to $\sigma_{\text{0}}^{\text{2}}$ and the ratio *λ*_2_ of $\sigma_{N}^{2}$ to $\sigma_{\text{0}}^{\text{2}}$.

Then based on the random utility maximization, given the values of all parameters, the chosen probability of route *k* is equal to

$$P_{k}^{rs}=P[V_{n,k}^{rs}+C_{n,k}^{rs}+\varepsilon_{n,k}^{rs}\geq \text{max}(V_{n,j}^{rs}+C_{n,j}^{rs}+\varepsilon_{n,j}^{rs};j\neq k,j\in A_{n}^{rs})]$$ (11)

where $A_{n}^{rs}$ is the routes set between OD pair *rs* for passenger *n*. With respect to current scale of the metro network, the largest size of the routes set $A_{n}^{rs}$ can be set as 10.

## Model estimation

### The Bayesian formulation to the CMNP model

Faced with multidimensional integrals of the multivariate normal densities especially for large routes set, MNP model is usually estimated by Bayesian formulation and Monte Carlo Markov Chain (MCMC) approach [36]. This paper mainly wants to exhibit how to transform CMNP model with structured covariance into Bayesian formula and introduce Cholesky Decomposition to descend the dimension of integral so that the computational time can be saved. After the dimension reduction process and integral domain transformation, the calculation of multidimensional integrals of the multivariate normal densities given the parameters can be carried out based on quasi-Monte Carlo method.

Here, we construct a vector **ζ** = **μ**∪σ∪Σ = **θ**∪**ω**∪**γ**∪**z**∪**λ** including all unknown parameters, where **θ** contains the parameters in the compensatory component; **ω** is the vector with the scale parameters; **γ** is the vector with the location parameters; **z** contains the parameters in the threshold function, that is *α*_*n*_ and *β*_*n*_ in this paper; **λ** covers the parameters in the covariance matrix. Meanwhile, the vector **Y** denotes the indicators of the observations referring to the chosen routes. Compared with the traditional probit model and the proposed CMNP model, the non-compensatory component in the CMNP model leads to the difference between the two models. However, the value of the non-compensatory component can be easily calculated given the unknown parameters, benefiting from the independent bivariate normal distribution assumption. Based on Bayes’ theorem, the posterior distribution *π*(**ζ**|**Y**) is proportional to the priors on all unknown parameters, that is, the joint posterior distribution for the Hierarchical Bayes model is as follows.

π(ζ|Y)∝P(Y|θ,ω,γ,z,λ)⋅π(θ,ω,γ,z,λ|ζ)⋅π(ζ) (12)

where *π*(·) is the probability density function; *P*(**Y**|**θ**, **ω**, **γ**, **z**, **λ**) is the probability of observation **Y** given all unknown parameters which is equal to Eq (11). Supposing that all parameters are independent from each other, we can get the below equations.

$$\pi (\theta ,\omega ,\gamma ,z,\lambda |\zeta )=\pi (\theta |\mu_{\theta },\sigma_{\theta })\cdot \pi (\omega |\mu_{\omega },\sigma_{\omega })\cdot \pi (\gamma |\mu_{\gamma },\sigma_{\gamma })\cdot \pi (z|\mu_{z},\sigma_{z})\cdot \pi (\lambda |\mu_{\lambda },\sigma_{\lambda })$$ (13)

$$\pi (\zeta )=\pi (\mu_{\theta })\cdot \pi (\sigma_{\theta })\cdot \pi (\mu_{\omega })\cdot \pi (\sigma_{\omega })\cdot \pi (\mu_{\gamma })\cdot \pi (\sigma_{\gamma })\cdot \pi (\mu_{z})\cdot \pi (\sigma_{z})\cdot \pi (\mu_{\lambda })\cdot \pi (\sigma_{\lambda })$$ (14)

Probit model is hard to be calculated even if all the unknown parameters are given because of the multivariate normal distribution. The Eq (11) can be rewritten into the D-dimensional integrals of the multivariate normal density as follows.

$$P_{k}^{rs}=\int_{\varepsilon_{1}^{rs}=-\infty }^{\varepsilon_{k}^{rs}+V_{k}^{rs}+C_{k}^{rs}-V_{1}^{rs}-C_{1}^{rs}}\cdots \int_{\varepsilon_{k}^{rs}=-\infty }^{\text{+}\infty }\cdots \int_{\varepsilon_{m}^{rs}=-\infty }^{\varepsilon_{k}^{rs}+V_{k}^{rs}+C_{k}^{rs}-V_{m}^{rs}-C_{m}^{rs}}\Omega (\varepsilon^{rs})d\varepsilon_{m}^{rs}\cdots d\varepsilon_{1}^{rs}$$ (15)

$$\Omega (\varepsilon^{rs})={\left(2\pi \right)}^{\frac{-m}{2}}|\Sigma {|}^{\frac{-1}{2}}\text{exp}[-\frac{1}{2}\varepsilon^{rs}\Sigma^{-1}{\left(\varepsilon^{rs}\right)}^{T}]$$ (16)

where $\varepsilon^{rs}=[\varepsilon_{1}^{rs},\varepsilon_{2}^{rs},\cdots ,\varepsilon_{m}^{rs}]$ is the random error vector.

Considering that the covariance matrix is a Hermitian, positive-definite matrix, the integral of the general multivariate normal distribution can be transformed into that of standard normal distribution via Cholesky Decomposition to the covariance matrix and other substitutions [37–38]. By this means, the integral domain is referred to as transforming the *m*-variate integral into one over the (*m*-1)-dimensional hypercube.

Based on Cholesky Decomposition, the covariance matrix can be written as

$$\Sigma =D\times D^{T}$$ (17)

where *D* is a lower triangular matrix and *D*^*T*^ is its conjugate transpose. We set

$$\varepsilon^{rs}=D\times q^{rs}$$ (18)

where $q^{rs}=[q_{1}^{rs},q_{2}^{rs},\cdots ,q_{m}^{rs}]$ is a vector substituting random error vector **ε**^*rs*^. Then the Eq (11) can be transformed as

$$P_{k}^{rs}={\left(2\pi \right)}^{\frac{-m}{2}}\int_{-\infty }^{{b^{\prime }}_{1}}\text{exp}(-\frac{q_{1}^{2}}{2})\cdots \int_{-\infty }^{{b^{\prime }}_{m}}\text{exp}(-\frac{q_{m}^{2}}{2})dq^{rs}$$ (19)

$${b^{\prime }}_{i}=\left\{ \begin{array}{l} (\sum_{j=1}^{k}d_{k,j}q_{j}+V_{k}^{rs}+C_{k}^{rs}-V_{i}^{rs}-C_{i}^{rs}-\sum_{j=1}^{i-1}d_{i,j}q_{j})/d_{i,i}\begin{array}{cc} , & i\neq k \end{array}\\ +\infty \begin{array}{cccc} & & , & i=k \end{array} \end{array}\right.$$ (20)

where ${b^{\prime }}_{i}$ is the upper limit of the *i*-th layer’s integration; *d*_*i*,*i*_ is the element in *D*. We assume

$$q_{i}=\Phi^{-1}(v_{i})$$ (21)

where $v^{rs}=[v_{1}^{rs},v_{2}^{rs},\cdots ,v_{m}^{rs}]$ is a vector substituting vector **q**^*rs*^; Φ^−1^(·) is the inverse cumulative probability function of standard normal distribution, that is *v*_*i*_ = Φ(*q*_*i*_). Meanwhile, we suppose *v*_*i*_ = *w*_*i*_*e*_*i*_. Thus, we get the equation

Pkrs=∫0e1⋯∫0ek⋯∫0emdvrs=∫01∫01⋯∫01∏i=1meidw (22)

$$e_{i}=\left\{ \begin{array}{l} \Phi ((\sum_{j=1}^{k}d_{k,j}\Phi^{-1}(w_{j}\cdot e_{j})+V_{k}^{rs}+C_{k}^{rs}-V_{i}^{rs}-C_{i}^{rs}-\sum_{j=1}^{i-1}d_{i,j}\Phi^{-1}(w_{j}\cdot e_{j}))/d_{i,i})\begin{array}{cc} , & i\neq k \end{array}\\ 1\begin{array}{cccc} & & & \begin{array}{cc} \begin{array}{cc} & , \end{array} & i=k \end{array} \end{array} \end{array}\right.$$ (23)

where **w** = (*w*_1_, *w*_2_,…,*w*_*m*_) denotes the parameters vector.

When *i* = *k*, *e*_*i*_ = 1, the *m*-dimensional integration declines into (*m*-1)-dimensional integration. And quasi-Monte Carlo method can be used to calculate the probability of multivariate normal distribution given all unknown parameters.

### The parameter identification problem

With respect to the constrained multinomial logit model, Castro et al. discussed the parameter identification problem derived from the fact that one attribute exists both in the compensatory and non-compensatory components [23]. This problem has been avoided in this paper via the process that the threshold is regarded as a function whose value varies with the change of the OD scale. By this means, the parameters associated with the same attribute both in the compensatory and non-compensatory components are identifiable. Another parameter identification problem arises when the threshold parameter plus directly location parameter. In this paper, the constraint of number of transfers suffers this problem as *β*+*γ*_*τ*_, where *β* is the threshold parameter and *γ*_*τ*_ is the location parameter. The two parameters cannot be identified, but we can estimate the sum of them without influence on other parameters. For simplicity, we can assume the location parameter is equal to 0, and then we can get the value of the threshold parameter.

### Probability calculation based on Monte Carlo simulation

With respect to the solution to Eq (11) associated with the probit probability, the quasi-Monte Carlo method is carried out. Supposing that every element in **w** follows the uniform distribution *w*_*i*_ ~ *U*(0, 1) and the elements in **w** are independent from each other, we use Halton sequence to generate random data. The vector **w**_*j*_ = (w_1*j*_,…*w*_*ij*_,…*w*_*mj*_). contains the random values generated by the Halton sequence in the *j*-th iteration. We can get the approximate solution to Eq (11) given unknown parameters **θ**, **ω**, **γ**, **z**, **λ**, that is,

$$\begin{array}{l} P_{k}^{rs}=E(\prod_{i=1}^{m}e_{i}(w))\\ \begin{array}{cc} & \approx \frac{1}{J}\sum_{j=1}^{J}\left(\prod_{i=1}^{m}e_{i}(w_{j})\right) \end{array} \end{array}$$ (24)

where *E*(·) denotes the expected value.

### Estimation algorithm

The Metropolis-Hastings (M-H) algorithm [39–40] which is known as one of the MCMC approach is widely used to generate samples of the parameter from a prior distribution without the prior knowledge. In order to generate the final samples, candidates are drawn iteratively and they will be accepted as current samples with a certain probability in every iteration. With the increase of the number of iterations, Markov Chain ensures that we will gain a stationary posterior distribution of the parameter. In order to improve estimating efficiency, the variable-at-a-time Metropolis sampling scheme [39] is used to generate candidate for every parameter in turn in the parameters’ set. The estimation process is organized as follows.

*Step* 1: Generate randomly initial values from pre-defined prior distribution, that is $\zeta^{\left(0\right)}=(\zeta_{1}^{\left(0\right)},\cdots ,\zeta_{i}^{\left(0\right)},\cdots \zeta_{NP}^{\left(0\right)})$, where *NP* denotes the number of parameters. And generate routes sets **A**^*rs*^ for OD pairs based on physical length. Set iteration *t* = 1 and *i* = 1.

*Step* 2: Draw a candidate $\zeta^{*}=(\zeta_{1}^{\left(t\right)},\cdots ,\zeta_{i-1}^{\left(t\right)},\zeta_{i}^{*},\zeta_{i+1}^{\left(t-1\right)},\cdots \zeta_{NP}^{\left(t-1\right)})$ from a jumping distribution $J_{t}(\zeta_{i}^{*}|\zeta_{i}^{\left(t-1\right)})$ based on the Gaussian random walk Metropolis sampling method. This method suggests that the jumping distribution is supposed to be a normal distribution which is a symmetric distribution satisfying the equation $J_{t}(\zeta_{i}^{*}|\zeta_{i}^{\left(t-1\right)})=J_{t}(\zeta_{i}^{\left(t-1\right)}|\zeta_{i}^{*})$. Here we set the jumping distribution as $J_{m}(\zeta_{i}^{*}|\zeta_{i}^{\left(m-1\right)})\sim N(\zeta_{i}^{\left(m-1\right)},\xi^{2})$, where *ξ*^2^ is the proposal variance for the *i-*th parameter.

*Step* 3: Calculate the acceptance ratio $\vartheta =\text{min}\{1,\frac{\pi^{\prime }(\zeta_{i}^{*})J_{m}(\zeta_{i}^{\left(m-1\right)}|\zeta_{i}^{*})}{\pi^{\prime }(\zeta_{i}^{\left(m-1\right)})J_{m}(\zeta_{i}^{*}|\zeta_{i}^{\left(m-1\right)})}\}=\text{min}\{1,\frac{\pi^{\prime }(\zeta_{i}^{*})}{\pi^{\prime }(\zeta_{i}^{\left(m-1\right)})}\}$, where

$$\begin{array}{ll} \pi^{\prime }(\zeta_{i}^{*}) & =\pi (\zeta_{1}^{\left(t\right)},\cdots ,\zeta_{i-1}^{\left(t\right)},\zeta_{i}^{*},\zeta_{i+1}^{\left(t-1\right)},\cdots \zeta_{NP}^{\left(t-1\right)}|Y)\\ & \propto \underset{k\in Y}{\prod }[P(k|\zeta_{1}^{\left(t\right)},\cdots ,\zeta_{i-1}^{\left(t\right)},\zeta_{i}^{*},\zeta_{i+1}^{\left(t-1\right)},\cdots \zeta_{NP}^{\left(t-1\right)})\cdot \overset{i-1}{\underset{j=1}{\prod }}\pi (\zeta_{j}^{\left(t\right)})\cdot \overset{NP}{\underset{j=i+1}{\prod }}\pi (\zeta_{j}^{\left(t-1\right)})\cdot \pi (\zeta_{i}^{*})] \end{array}$$ (25)

$$\begin{array}{ll} \pi^{\prime }(\zeta_{i}^{\left(t-1\right)}) & =\pi (\zeta_{1}^{\left(t\right)},\cdots ,\zeta_{i-1}^{\left(t\right)},\zeta_{i}^{\left(t-1\right)},\zeta_{i+1}^{\left(t-1\right)},\cdots \zeta_{NP}^{\left(t-1\right)}|Y)\\ & \propto \underset{k\in Y}{\prod }[P(k|\zeta_{1}^{\left(t\right)},\cdots ,\zeta_{i-1}^{\left(t\right)},\zeta_{i}^{\left(t-1\right)},\zeta_{i+1}^{\left(t-1\right)},\cdots \zeta_{NP}^{\left(t-1\right)})\cdot \overset{i-1}{\underset{j=1}{\prod }}\pi (\zeta_{j}^{\left(t\right)})\cdot \overset{NP}{\underset{j=i+1}{\prod }}\pi (\zeta_{j}^{\left(t-1\right)})\cdot \pi (\zeta_{i}^{\left(t-1\right)})] \end{array}$$ (26)

where $P(k|\zeta_{1}^{\left(t\right)},\cdots ,\zeta_{NP}^{\left(t-1\right)})=P_{k}^{rs}$, referring to Eq (11). The same parts can be canceled out and the ratio is

$$\vartheta =\text{min}\{1,\frac{\prod_{k\in Y}\left[P(k|\zeta_{1}^{\left(t\right)},\cdots ,\zeta_{i-1}^{\left(t\right)},\zeta_{i}^{*},\zeta_{i+1}^{\left(t-1\right)},\cdots \zeta_{NP}^{\left(t-1\right)}\right)\cdot \pi (\zeta_{i}^{*})]}{\prod_{k\in Y}\left[P(k|\zeta_{1}^{\left(t\right)},\cdots ,\zeta_{i-1}^{\left(t\right)},\zeta_{i}^{\left(t-1\right)},\zeta_{i+1}^{\left(t-1\right)},\cdots \zeta_{NP}^{\left(t-1\right)}\right)\cdot \pi (\zeta_{i}^{\left(m-1\right)})]}\}$$ (27)

*Step* 4: Draw a value *u* from the uniform distribution *U*(0, 1). If *u*≤*ϑ*, **ζ**^(*m*)^ = ζ*; otherwise, **ζ**^(*m*)^ = **ζ**^(*m*−1)^.

*Step* 5: If *i*<*NP*, *i* = *i*+1, repeat *Step* 2—*Step*4; otherwise, continue *Step* 6.

*Step* 6: If *m*<*M*, *m* = *m*+1, *i* = 1, repeat *Step* 2—*Step* 5; otherwise, stop sampling.

## Results and discussions

### Data

All data are available in the supporting information file (S1_Dataset.).

With new lines put into operation ceaselessly, Guangzhou Metro becomes the sixth busiest metro system in the world and the third largest metro system in China. Up to July 2014, there are 8 lines and 136 stations (including 19 transfer stations) in operation, forming 256.6km operating length and carrying about 6.2 million daily ridership, except the APM Line. The APM Line isn’t in our consideration, because it belongs to a unique system which needs to swipe through again although you were in other lines. The metro system covers the major urban areas of the city, reaches into some large suburban area and connects Guangzhou city and Foshan city. Through statistical analysis, there are 3721 OD pairs for which the routes with the shortest travel times are not the routes with the minimum number of transfers. It provides the possibility that more than one route will be considered by passengers with comprehensive consideration of multiple factors. The large scaled metro network increases the complexity of route choice analysis.

**Fig 3:**
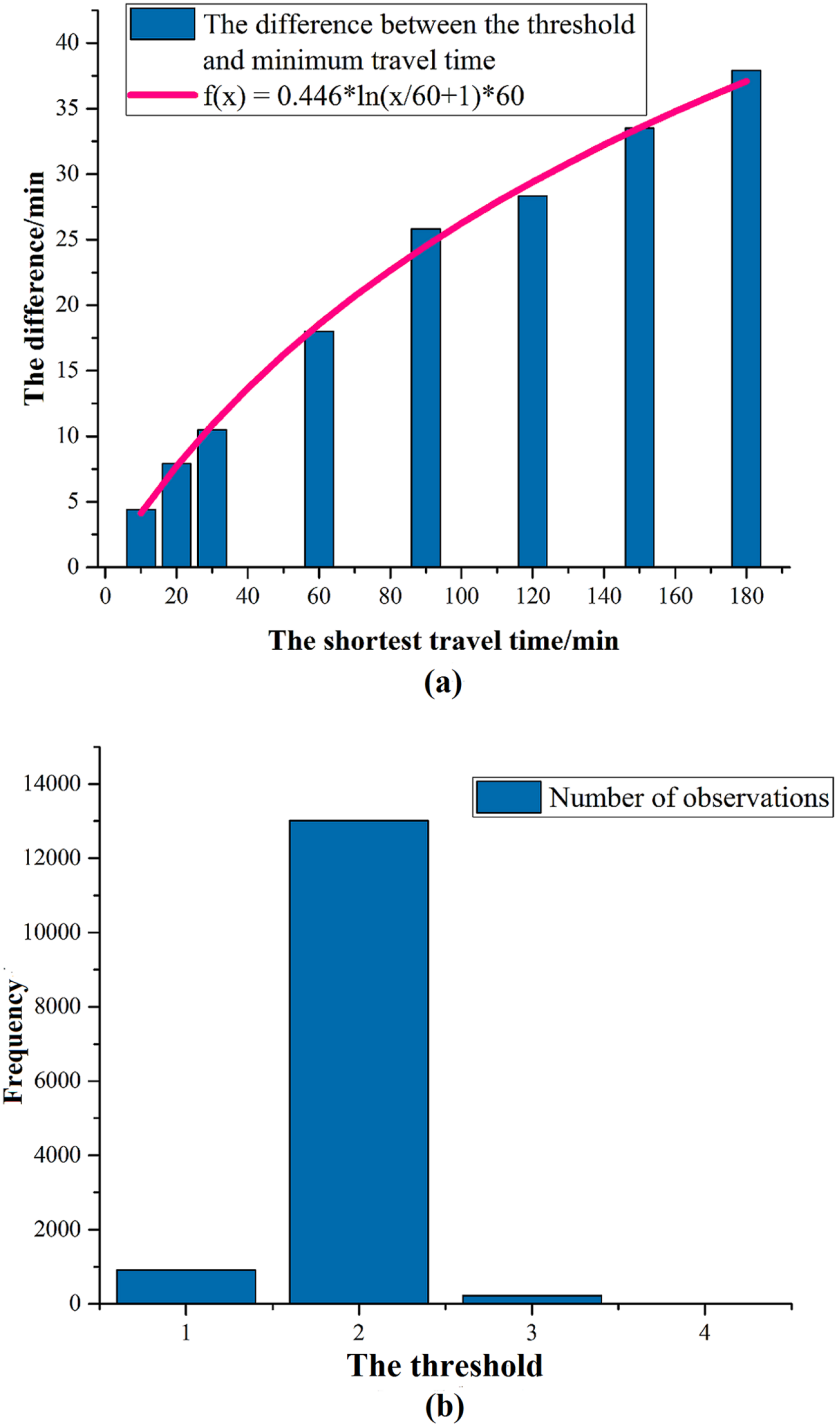
The thresholds of travel time and number of transfers. The figure displays the survey data associated with thresholds. By fitting, the parameters in the thresholds can be estimated.

In July 2014, Guangzhou Metro Corporation organized a survey in the metro stations to collect passengers’ travel characteristics, such as respondents’ actual travel routes. Totally, the effective sample size is 14142. Based on the survey data, Fig 3(a) shows the relationship between the difference (namely the threshold minus the shortest travel time) and the shortest travel time. It can be seen that the difference increases logarithmically with the increase of the shortest travel time which demonstrates that the travel time threshold formula in Eq (7) is suitable. By data fitting, when *α*_*n*_ = 0.446, the mean absolute percentage error (MAPE) is the minimum 3.657%. Fig 3(b) shows that when the minimum number of transfers is 0, the weighted mean value of the threshold of number of transfers is 1.951, that is, *β*_*n*_ can be assumed as 1.951.

### Estimations

In the compensatory component, the in-vehicle travel time ($X_{k,\text{1}}^{rs}$, h), number of transfers ($X_{k,\text{2}}^{rs}$, time), transfer time ($X_{k,\text{3}}^{rs}$, h), comfort degree ($X_{k,\text{4}}^{rs}$, 0–1 variable) and revised angular cost ($X_{k,\text{5}}^{rs}$, km) are considered with the corresponding parameters *θ*_1_, *θ*_2,_ *θ*_3,_ *θ*_4_ and *θ*_5_, where revised angular cost $X_{k,\text{5}}^{rs}$ measures the deviation degree of a route by transforming *sin()* into *tan()* and comfort degree $X_{k,\text{4}}^{rs}$ represents the congestion level in the train whose value is $X_{k,\text{4}}^{rs}=1$ when average load factor of one route is smaller than 20%, otherwise, $X_{k,\text{4}}^{rs}=0$. In the non-compensatory component, the travel time ($X_{k,\text{0}}^{rs}=X_{k,\text{1}}^{rs}+X_{k,\text{3}}^{rs}$$X_{k,\text{3}}^{rs}$, h) and number of transfers $X_{k,\text{2}}^{rs}$ are considered with the threshold parameters *α* and *β*, scale parameters *ω*_*t*_ and *ω*_*m*_ as well as location parameters *γ*_*t*_ and *γ*_*m*_ respectively. In case of the parameter identification problem in the threshold of number of transfers, the location parameter is assumed to be 0, that is *γ*_*m*_ = 0. Moreover, the parameter *λ*_1_ and *λ*_2_ in the covariance matrix needs to be estimated.

**Fig 4:**
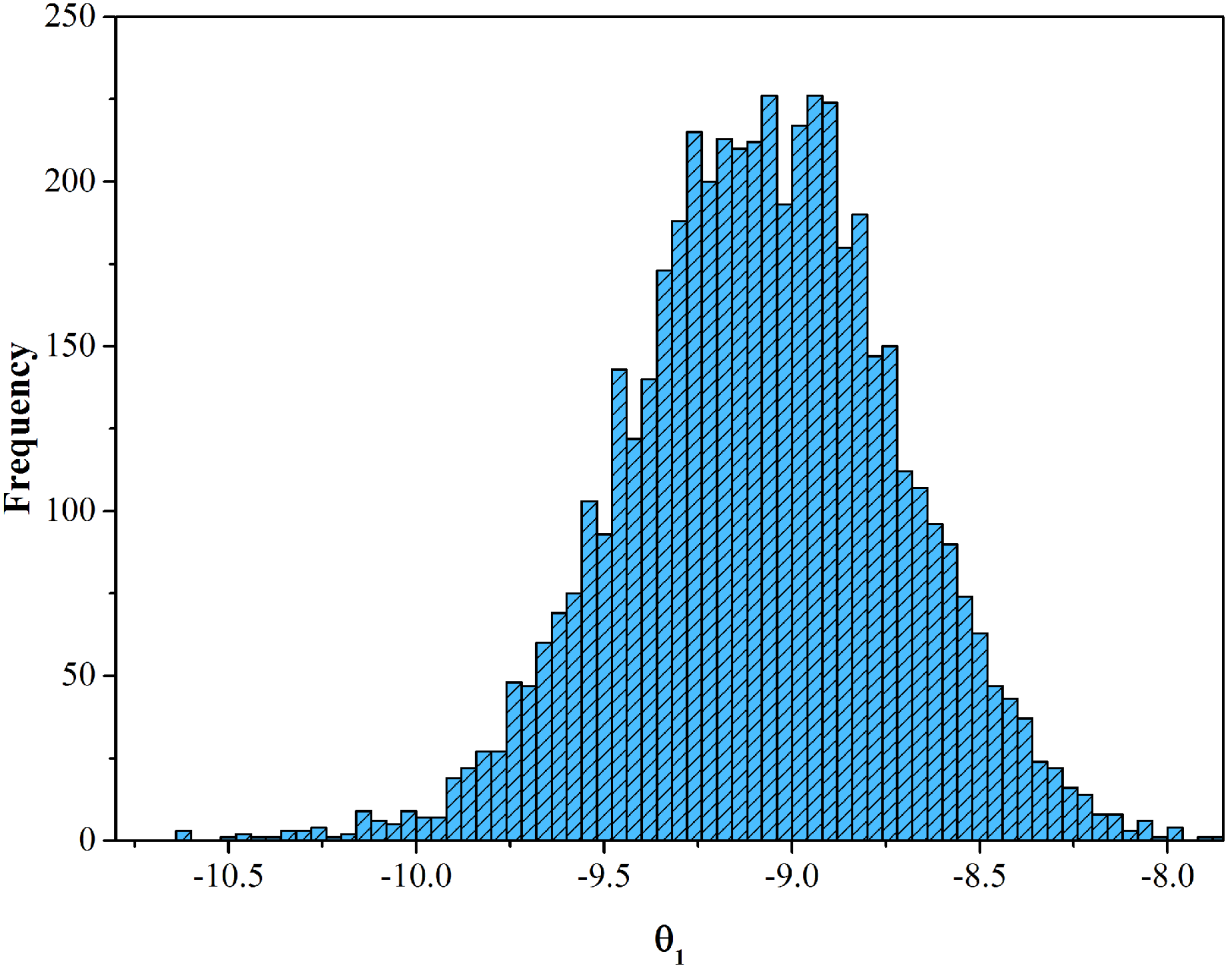
The distribution of effective samples for *θ*_1_. The figure exhibits the frequency distribution of the samples to represent the convergence directly.

Based on the surveyed data, the proposed model, MNP model, MNL model and CMNL model are estimated respectively. The latter two models are estimated based on the maximization likelihood estimation method, while the proposed model and MNP model are estimated based on the estimation approach proposed by this paper. Under the non-informative condition, the prior distributions for all parameters are assumed to follow uniform distribution. Totally, what parameters we need to estimate are *θ*_1_, *θ*_2_, *θ*_3_, *θ*_4_, *θ*_5_, *α*, *β*, *ω*_*t*_, *ω*_*m*_, *γ*_*t*_, *λ*_1_ and *λ*_2_. Considering the signs of the parameters, we assume that *θ*_1_, *θ*_2_, *θ*_3_ and *θ*_5_ follow U(-20, 0); *θ*_4_, *α*, *β*, *ω*_*m*_, *λ*_1_ and *λ*_2_ follow U(0, 20); *ω*_*t*_ follows U(60, 200); *γ*_*t*_ follows U(-1, 1). The proposal variance *ξ*^2^ is 0.05. The surveyed RP data is divided into two parts, referring to the 12039 data for estimation and 2103 data for examination. The scheme to screen the data will be described later. Based on MCMC approach, we tried 10000 iterations to estimate all parameters in the CMNP model, where the fore 5000 samples for each parameter are abandoned as burn-in period and the left 5000 effective samples for each parameter are drawn. The distribution of the effective samples for *θ*_1_ is taken as an example shown in Fig 4. Based on the samples, we can get the mean and 95% Bayesian conference interval (CI) shown in Table 1, as well as the estimations of MNL, MNP and CMNL models.

**Table 1:** Estimations of MNL, CMNL and CMNP models.

Parameter	MNL-value (*t*-value)	MNP-mean (95% CI)	CMNL-value (*t*-value)	CMNP-mean (95% CI)
*θ*_1_	-14.411 (-31.103)	-13.174 ([-13.165, -13.184])	-10.224 (-40.714)	-9.074 ([-9.065, -9.084])
*θ*_2_	-2.256 (-17.267)	-2.071 ([-2.061, -2.081])	-1.641 (-15.324)	-1.479 ([-1.468, -1.489])
*θ*_3_	-15.623 (-16.654)	-13.111 ([-13.100, -13.121])	-11.712 (-13.125)	-11.100 ([-11.088, -11.111])
*θ*_4_	0.005 (6.068)	0.008 ([0.00799,0.00801])	0.004 (3.224)	0.008 ([0.00799,0.00801])
*θ*_5_	-0.018 (-2.016)	-0.052 ([-0.0520, -0.0523])	-0.061 (-2.514)	-0.075 ([-0.0751, -0.0753])
*α*	--	--	0.460 (3.412)	0.455 ([0.450, 0.460])
*β*	--	--	1.961 (2.121)	1.959([1.943, 1.975])
*ω*_*t*_	--	--	65.012 (21.403)	177.392 ([177.240, 177.548])
*ω*_*m*_	--	--	6.051 (5.711)	7.431 ([7.411, 7.452])
*γ*_*t*_	--	--	0.001 (1.991)	0.001 ([0.0009, 0.0011])
*λ*_*1*_	--	0.621([0.620, 0.622])	--	0.675([0.674, 0.676])
*λ*_*2*_	--	0.544([0.542, 0.546])	--	0.552([0.550, 0.554])
*ρ*^*2*^	0.442	0.571	0.533	0.723
Sample Size	10000	10000	10000	10000

Kolmogorov-Smirnov (KS) Test is used to determine whether the samples follow normal distribution. Fig 4 shows the distribution of the effective samples for parameter *θ*_1_ for an example. Descriptive statistics show that the average value is -9.074 and the standard deviation is 0.366. By KS test, the *p* value is 0.433 which is greater than 0.05, proving that the samples follow normal distribution at 5% significance level. Thus, the sampling process converges. Other parameters have the same characteristics. As shown in Table 1, the different results for the parameters in MNP and CMNP models indicate that the MCMC approach can successfully distinguished all parameters though some of them have the same initial values. And the CI for every parameter ensures us to accept the means of the drawn samples. Meanwhile, coefficients of in-vehicle time, number of transfers, transfer time and revised angular cost are negative, meaning that the chosen probability of one route decreases along with the increase of in-vehicle time, number of transfers, transfer time or revised angular cost. Coefficients of comfort degree in both models are all positive, meaning that their increase will improve individual preference to the route. It is consistent with the common sense. Furthermore, *t*-values of the coefficients for MNL and CMNL models exceed 1.96, indicating that the null hypothesis that the true values of the coefficients are zero can be rejected at the 0.05 significance level. And the *ρ*^2^ of all models are greater than 0.2, indicating that all models have a good goodness-of-fit. Compared with the *ρ*^2^, CMNP model are the greatest, illustrating that the proposed CMNP model is the best among all models.

**Fig 5:**
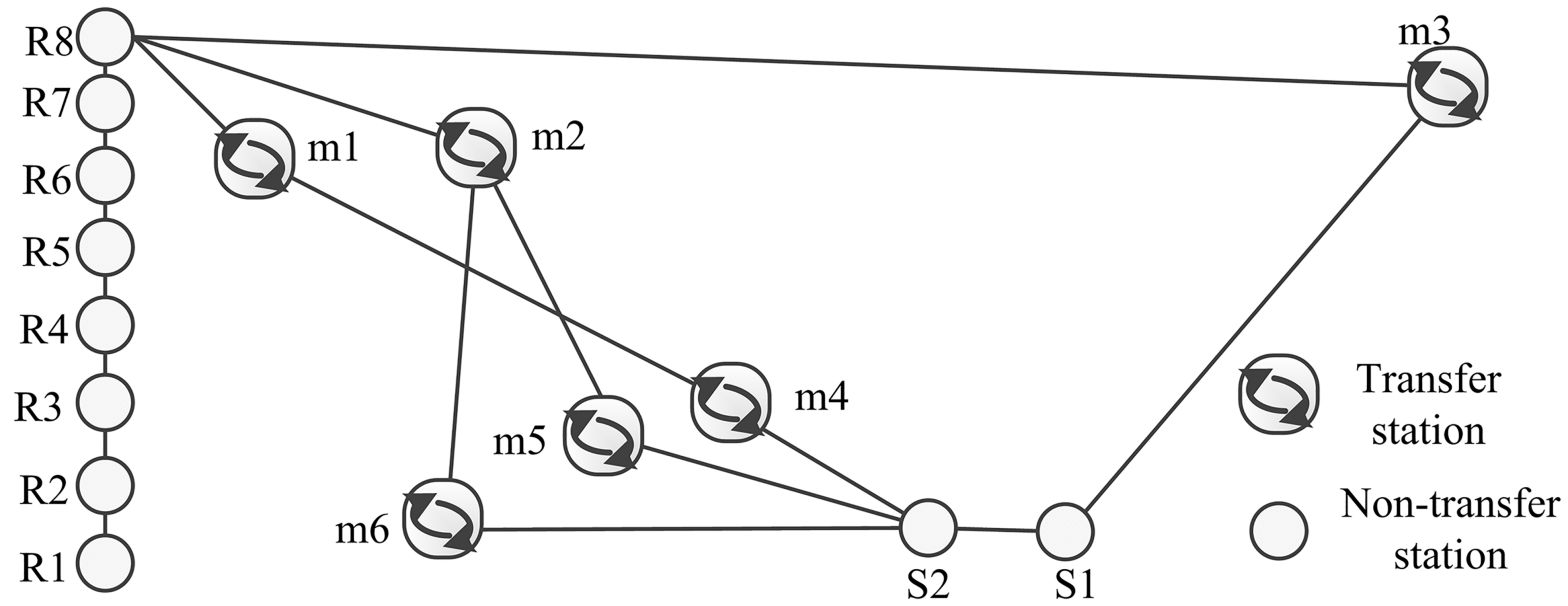
A diagram of deleted OD pairs and routes. The origin stations are denoted as R; the destination stations are denoted as S; the transfer stations are denoted as m. Other stations are omitted in the figure. It means transferring if the route passes by a transfer station.

In addition to the estimation performance, the forecasting performances of all models are compared. In order to gain plenty of actual choice results which are drawn from the surveyed data, the route choices between some similar OD pairs are aggregated. As shown in Fig 5, we combine the origins as an identical origin **R** as well as the identical destination **S**, that is, **R** contains Guangzhou South Railway Station (R1), Shibi (R2), Huijiang (R3), Nanpu (R4), Luoxi (R5), Nanzhou (R6), Dongxiao South (R7) and Jiangtai Road (R8); **S** contains Jingxi Nanfang Hospital (S1) and Meihuayuan (S2). The transfer stations are Haizhu Square (*m*1), Gongyuanqian (*m*2), Jiahewanggang (*m*3), Yantang (*m*4), Guangzhou East Railway Station (*m*5) and Tiyu West Road (*m*6). Excluding the routes with chosen probabilities smaller than 0.0001, we have four routes left in Table 2 along with the chosen probabilities according to different models. The number of the actual choices between the specific OD pairs in the surveyed data is 2103 and the absolute error is calculated to compare the forecasting performance as shown in Table 2. We can see that CMNP model has the smallest MAE (Mean Absolute Error) which demonstrates that the proposed CMNP model has the best forecasting performance.

**Table 2:** The comparisons of all models.

Route	Actual Prob.	MNL Prob.(AE)	MNP Prob.(MAE)	CMNL Prob.(AE)	CMNP Prob.(AE)
*K*1: R -> *m*2 -> *m*6 -> S	51%	45% (6%)	46% (5%)	58% (7%)	53% (2%)
*K*2: R -> *m*2 -> *m*5 -> S	19%	38% (19%)	27% (8%)	23% (4%)	21% (2%)
*K*3: R -> *m*3 -> S	21%	13% (8%)	16% (5%)	12% (9%)	20% (1%)
*K*4: R -> *m*1 -> *m*4 -> S	9%	4% (5%)	11% (2%)	7% (2%)	6% (3%)
Sum	100%	100% (38%)	100% (20%)	100% (22%)	100% (8%)

### Application

**Fig 6:**
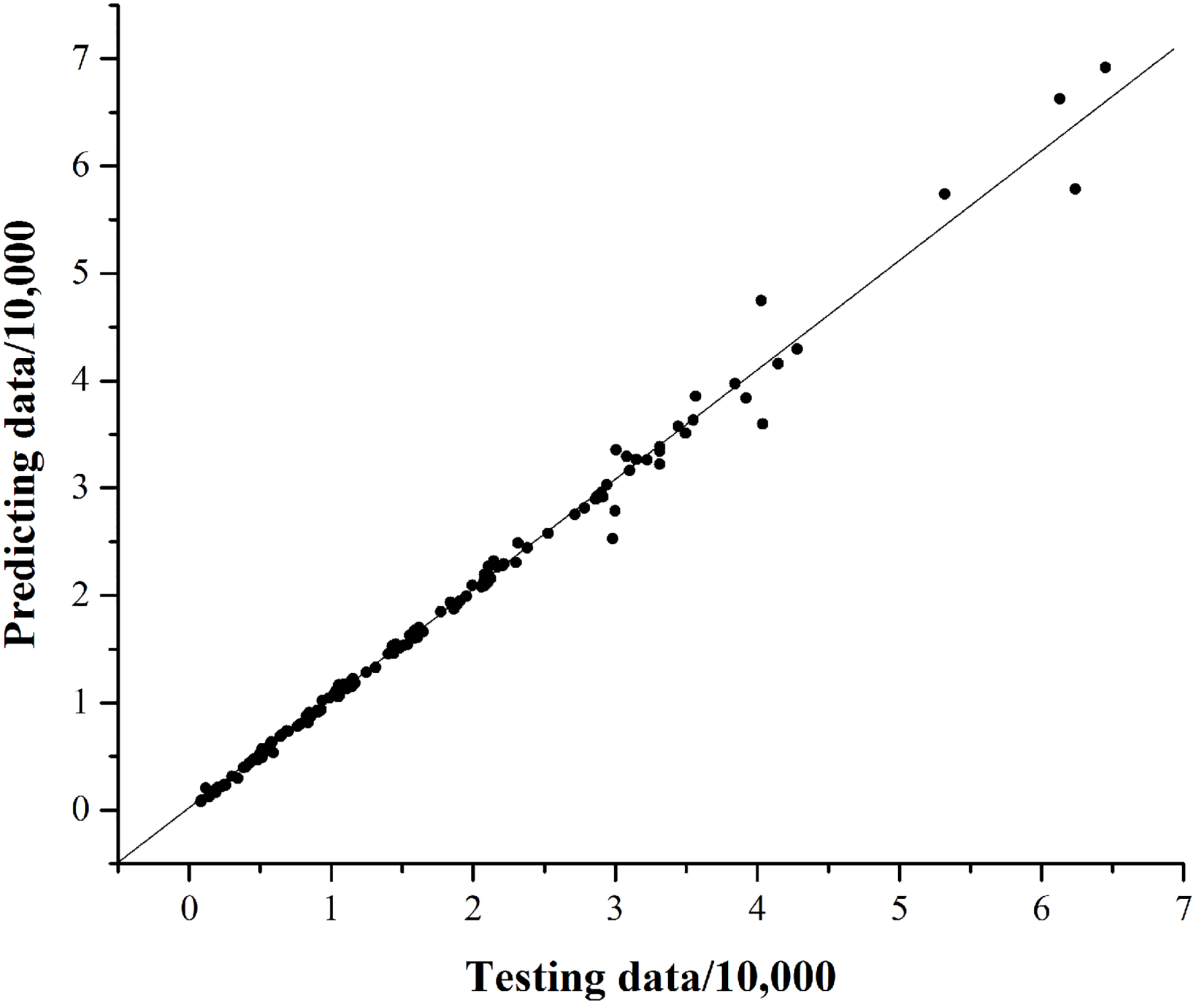
The transfer flow volume forecasting performance. For each spot, it has two values, including the testing data corresponding to horizontal axis and forecasting value corresponding to vertical axis.

The route choice model can be used to predict the transfer flow volume, section flow volume, etc. which are the basis of scheduling the train plan, guiding individual travel route, etc. The proposed CMNP route choice model determines the route choice probability for every OD pair in the metro network. And then the flow volume on the route can be derived from the product of the probability and the OD volume. By counting the number of passengers transferring between two different lines based on the train timetable, the transfer flow volume can be calculated. All testing data are provided by Guangzhou Metro Corporation. The results are shown in Fig 6 where testing data is on the horizontal axis, predicting data is on the vertical axis, and the solid line is the basic line indicating that the predicting data is equal to the testing data if the data spot is on the line. Every spot represents the flow volume transferring form one running direction of one line to one running direction of another line. Usually, every line has two running directions. The mean absolute percentage error (MAPE) is 4.91% which shows that the proposed CMNP model has a good application prospect.

## Conclusion

In a large scaled metro network, the complex nature of route choice process brings us a challenge to exactly figure out passengers’ actual decision rules. This paper focuses on integrating the impacts of routes set and the interdependency among alternative routes on route choice probability into route choice modeling in the metro network. The impact of routes set on route choice probability expresses the semi-compensatory choice process which is a combination of routes set generation and route choice stages. Thereafter, a constrained multinomial probit (CMNP) model is proposed by this paper, in which, the utility function consists of compensatory, non-compensatory and error parts. The compensatory part is a linear function of in-vehicle travel time, number of transfers, transfer time, congestion level and revised angular cost. The non-compensatory part measures the impact of considered probability of one route on the route’s utility by a logarithm function, where considered probability is calculated by a binary probit equation denoting the relationship between the constrained attributes (e.g. travel time and number of transfers) and the corresponding thresholds proposed by this paper. The error part follows a multivariate normal distribution, whose variance is structured into three parts, including measuring the correlation among routes, representing the transfer variance of the route, and denoting the unobserved variance.

With respect to the estimation, considering multidimensional integrals of the multivariate normal probability density function, the CMNP model is rewritten as Bayesian formulation and MCMC approach is constructed to estimate all parameters. As a key point to calculate the acceptance rate, given the unknown parameters, the multidimensional integrals of the multivariate normal probability density function can be transformed into those of standard normal distribution via Cholesky Decomposition to the covariance matrix and other substitutions. Then the integrals can be easily simulated by quasi-Monte Carlo algorithm.

At last, the proposed model is estimated by the proposed estimation approach based on the surveyed RP data in Guangzhou Metro. The estimations show that every parameter can be distinguished though they have the same initial values. And the Bayesian CI indicates the reliability of the mean of the samples. Moreover, compared with MNL, MNP and CMNL models, the proposed CMNP model shows the best forecasting performance with respect to the prediction on the route choice probabilities and transfer flow volumes.

In the future, we will try to estimate the proposed model based on the smart card data and the travel time reliability will also be considered in the model.

## Supporting information

S1 Table Estimations of MNL, CMNL and CMNP models. (DOCX)

S2 Table The comparisons of all models. (DOCX)

S1 Dataset All data used in this paper. (XLSX)
