# Supplementary material for: A constrained multinomial Probit route choice model in the metro network: Formulation, estimation and application
Source: PLoS One. 2017 Jun 7;12(6):e0178789. doi: 10.1371/journal.pone.0178789 (PMC5462412; doi:10.1371/journal.pone.0178789)
Supplement: S1 Table — (DOCX) [file pone.0178789.s001.docx]

Table 1. Estimations of MNL, CMNL and CMNP models.

| Parameter | MNL-value  (*t*-value) | MNP-mean  (95% CI) | CMNL-value  (*t*-value) | CMNP-mean  (95% CI) |
| --- | --- | --- | --- | --- |
| *θ*_1_ | -14.411  (-31.103) | -13.174  ([-13.165, -13.184]) | -10.224  (-40.714) | -9.074  ([-9.065, -9.084]) |
| *θ*_2_ | -2.256  (-17.267) | -2.071  ([-2.061, -2.081]) | -1.641  (-15.324) | -1.479  ([-1.468, -1.489]) |
| *θ*_3_ | -15.623  (-16.654) | -13.111  ([-13.100, -13.121]) | -11.712  (-13.125) | -11.100  ([-11.088, -11.111]) |
| *θ*_4_ | 0.005  (6.068) | 0.008  ([0.00799,0.00801]) | 0.004  (3.224) | 0.008  ([0.00799,0.00801]) |
| *θ*_5_ | -0.018  (-2.016) | -0.052  ([-0.0520, -0.0523]) | -0.061  (-2.514) | -0.075  ([-0.0751, -0.0753]) |
| *α* | -- | -- | 0.460  (3.412) | 0.455  ([0.450, 0.460]) |
| *β* | -- | -- | 1.961  (2.121) | 1.959([1.943, 1.975]) |
| *ω_t_* | -- | -- | 65.012  (21.403) | 177.392  ([177.240, 177.548]) |
| *ω_m_* | -- | -- | 6.051  (5.711) | 7.431  ([7.411, 7.452]) |
| *γ_t_* | -- | -- | 0.001  (1.991) | 0.001  ([0.0009, 0.0011]) |
| *λ_1_* | -- | 0.621([0.620, 0.622]) | -- | 0.675([0.674, 0.676]) |
| *λ_2_* | -- | 0.544([0.542, 0.546]) | -- | 0.552([0.550, 0.554]) |
| *ρ^2^* | 0.442 | 0.571 | 0.533 | 0.723 |
| Sample Size | 10000 | 10000 | 10000 | 10000 |
