# Supplementary material for: A constrained multinomial Probit route choice model in the metro network: Formulation, estimation and application
Source: PLoS One. 2017 Jun 7;12(6):e0178789. doi: 10.1371/journal.pone.0178789 (PMC5462412; doi:10.1371/journal.pone.0178789)
Supplement: S2 Table — (DOCX) [file pone.0178789.s002.docx]

Table 2. The comparisons of all models.

| Route | Actual  Prob. | MNL  Prob.(AE) | MNP  Prob.(MAE) | CMNL  Prob.(AE) | CMNP  Prob.(AE) |
| --- | --- | --- | --- | --- | --- |
| *K*1: R -> *m*2 -> *m*6 -> S | 51% | 45% (6%) | 46% (5%) | 58% (7%) | 53% (2%) |
| *K*2: R -> *m*2 -> *m*5 -> S | 19% | 38% (19%) | 27% (8%) | 23% (4%) | 21% (2%) |
| *K*3: R -> *m*3 -> S | 21% | 13% (8%) | 16% (5%) | 12% (9%) | 20% (1%) |
| *K*4: R -> *m*1 -> *m*4 -> S | 9% | 4% (5%) | 11% (2%) | 7% (2%) | 6% (3%) |
| Sum | 100% | 100% (38%) | 100% (20%) | 100% (22%) | 100% (8%) |
